# Effectiveness of evidence-based nursing interventions in the management of patients with schizophrenia

**DOI:** 10.3389/fpsyt.2025.1610260

**Published:** 2025-08-01

**Authors:** Jiao Wen, Ming-Yan Li, Pan-Pan Song, Fei Teng

**Affiliations:** ^1^ Outpatient Department, Shandong Mental Health Center, Jinan, Shandong, China; ^2^ Outpatient Department, Chaoyang No. 7 Retired Cadre Recuperation Center, Beijing, China; ^3^ Department of General Surgery, China-Japan Friendship Hospital, Beijing, China; ^4^ Department of Psychiatry III, Shandong Mental Health Center, Jinan, Shandong, China

**Keywords:** schizophrenia, evidence-based nursing, social functionality, psychiatric symptoms, medication adherence

## Abstract

**Background:**

Schizophrenia is a severe psychiatric disorder characterized by persistent symptoms, functional impairment, and a high risk of relapse. Evidence-based nursing (EBN) is a patient-centered approach that applies clinical research to improve treatment adherence, reduce symptom severity, and support recovery. This study aimed to evaluate the effectiveness of EBN interventions in improving clinical and functional outcomes in patients with schizophrenia.

**Material and methods:**

This retrospective study, conducted from January 2021 to December 2023, included 156 patients diagnosed with schizophrenia based on DSM-5 or ICD-10 criteria. Patients were divided into an observation group (n = 80) receiving EBN interventions and a control group (n = 76) receiving standard nursing care. EBN protocols included psychoeducation, behavioral rehabilitation, social skills training, family support, and medication supervision. Outcome measures included the Social Disability Screening Schedule (SDSS), Brief Psychiatric Rating Scale (BPRS), Modified Rehabilitation Status Scale (MRSS), and medication adherence rates. Statistical analyses were performed using SPSS 27.0, with a p-value of <0.05 indicating significance.

**Results:**

Baseline characteristics were comparable between groups. After intervention, the observation group demonstrated significant improvements in SDSS and BPRS scores compared to the control group, reflecting enhanced social functionality and symptom management (p < 0.001). MRSS indicators, including dependency, social function, activity ability, and symptom behavior, also showed greater improvements in the observation group (p < 0.001). Medication adherence was significantly higher in the observation group, with higher complete adherence rates (40.0% vs. 19.7%) and fewer cases of non-adherence (25.0% vs. 47.4%, p = 0.003).

**Conclusions:**

Evidence-based nursing interventions improve social functionality, symptom management, recovery states, and medication adherence in schizophrenia, emphasizing their value in optimizing clinical outcomes.

## 1 Introduction

Schizophrenia is a chronic and debilitating psychiatric disorder affecting over 20 million people globally. It is characterized by disturbances in thought, perception, emotion, language, and behavior, and is frequently accompanied by marked impairments in social and occupational functioning. The global burden of schizophrenia extends beyond individual and familial suffering, placing substantial strain on healthcare systems due to recurrent hospitalizations, long-term treatment demands, and complex care requirements. Given the chronic nature and severity of symptoms, comprehensive and effective management strategies are essential to support functional recovery. This highlights the need for structured, evidence-informed care models that address both the physiological and psychosocial dimensions of the disorder (1–3).

Evidence-based nursing (EBN) has become a pivotal approach in the management of complex psychiatric disorders such as schizophrenia. By integrating the best available evidence, clinical expertise, and patient values, EBN supports individualized, outcome-driven care. In schizophrenia, where symptom trajectories are variable and patient needs are multifaceted, EBN offers a structured framework to enhance treatment adherence, symptom control, and quality of life (4, 5). Furthermore, EBN enables nurses to make evidence-informed decisions that enhance intervention efficacy, reduce psychotic relapse, and support long-term recovery (6–9). However, despite promising outcomes reported in prior studies, most existing research focuses on isolated components of EBN or short-term symptom improvement, with limited evidence on its integrated application in psychiatric inpatient settings (10, 11). Furthermore, few studies in low- to middle-resource settings have systematically evaluated the impact of full-spectrum EBN interventions on functional recovery and medication adherence in patients with schizophrenia (12, 13). To address these gaps, this study aimed to assess the effectiveness of a comprehensive, evidence-based nursing model in improving clinical and functional outcomes in this population.

In this study, the EBN intervention was designed based on the Iowa Model of Evidence-Based Practice, which guides clinical decision-making through systematic problem identification, evidence appraisal, and team-based implementation. Unlike standard care, which typically includes medication guidance and periodic health education, our EBN protocol integrated structured psychoeducation, behavioral rehabilitation, family involvement, and medication supervision, each supported by empirical studies demonstrating improvements in psychiatric stability and social functioning. This structured, multidisciplinary approach was tailored to the inpatient psychiatric setting and aimed to provide continuity, individualization, and evidence alignment in nursing care. Through a comprehensive analysis of EBN’s effectiveness in schizophrenia management, this study seeks to evaluate its impact on clinical outcomes, patient satisfaction, and quality of life. By providing empirical evidence, this research aims to bridge existing gaps and encourage the broader adoption of EBN practices to improve care delivery and outcomes for individuals with schizophrenia.

## 2 Methods

### 2.1 Study design

This retrospective study, conducted at our hospital from January 2021 to December 2023, aimed to evaluate the effectiveness of EBN interventions in managing patients with schizophrenia. Standard nursing care was delivered from January 2021 to June 2022. Beginning in July 2022, the hospital formally implemented an EBN model as part of a departmental practice improvement initiative. Accordingly, patients hospitalized prior to July 2022 were assigned to the control group, while those admitted from July 2022 to December 2023 and managed under EBN protocols were included in the observation group. Patients eligible for inclusion had a confirmed diagnosis of schizophrenia according to DSM-5 or ICD-10 criteria and provided informed consent. Exclusion criteria included comorbid psychiatric disorders, severe cognitive impairments, and acute or unstable medical conditions that could interfere with participation. Additionally, patients with recent changes in primary antipsychotic medication within the last 8 weeks were excluded to minimize confounding effects on symptom evaluation. A total of 156 patients met the inclusion criteria. Of these, 76 patients receiving standard care were assigned to the control group, while 80 patients receiving EBN interventions comprised the observation group. The study was designed and reported in alignment with STROBE (Strengthening the Reporting of Observational Studies in Epidemiology) guidelines (14) Informed consent was obtained from all subjects. The study protocol was reviewed and approved by the hospital’s ethics committee. All procedures complied with relevant regulations and the ethical principles outlined in the Declaration of Helsinki. Participant data were anonymized and handled with strict confidentiality to ensure privacy.

### 2.2 Standard nursing care intervention protocols

Patients in the control group received standard nursing care, which included medication guidance, quarterly educational lectures on psychiatric health, and distribution of mental health booklets and informative materials. For patients experiencing negative emotions, psychological support was provided, emphasizing the importance of adherence to prescribed medications.

### 2.3 Evidence-based nursing interventions protocols

The observation group received a structured evidence-based nursing intervention program, which included the following components (15–17):

Formation of an EBN Team: A specialized team was formed, comprising six members, including the head nurse, senior nurses, and an associate chief physician. The team members underwent training on EBN principles and techniques, with clear role assignments. Together, they formulated and implemented the EBN care plan for schizophrenia management. The head nurse, as the lead of the EBN team, was responsible for overall care coordination, ensuring the implementation of evidence-based practices, and leading the multidisciplinary discussions. Senior nurses played a pivotal role in clinical decision-making, based on their advanced psychiatric nursing expertise, and in ensuring the consistency of care across all interventions.

Identifying Core Issues: Based on clinical experience and patient assessments, the team identified social functioning deficits as a primary issue in patients with schizophrenia. The main contributing factors included inadequate disease awareness, inconsistent medication adherence, and medication side effects impacting compliance. Nurses conducted thorough initial and ongoing assessments of patients’ psychosocial status, using standardized tools such as the Social Disability Screening Schedule (SDSS) and the Brief Psychiatric Rating Scale (BPRS) to identify deficits in social functioning, medication adherence, and mental health. They were also responsible for identifying patients’ concerns, barriers to treatment, and providing ongoing feedback to the EBN team.

Evidence-Based Support: The team conducted a comprehensive review of authoritative literature to ensure a reliable foundation for the nursing plan. Clinical data and prior experience guided the development of targeted, evidence-supported nursing strategies. Nurses in collaboration with the associate chief physician, actively reviewed the most current evidence in psychiatric nursing care and integrated these findings into the care plans. They ensured the nursing interventions adhered to the highest standards of evidence-based practice, enhancing the quality and effectiveness of care.

Nursing Measures:

1. Psychological Interventions: The psychological state of each patient was assessed to identify concerns and address anxiety, irritability, or other adverse emotions. Patients were educated on the mechanisms of schizophrenia, treatment approaches, and the importance of adherence, aiming to reduce psychological barriers. Psychiatric nurses independently assessed each patient’s psychological state, identifying specific emotional or cognitive challenges. They provided psychoeducation on schizophrenia and treatment, emphasizing the importance of adherence to medication. Additionally, nurses facilitated individualized therapeutic conversations, offering emotional support and reducing psychological barriers.
2. Behavioral and Skill Rehabilitation: Tailored programs included activities such as drawing, calligraphy, and music therapy, ensuring at least 15 hours of skill-based training per week. Additionally, vocational counseling and simulated work tasks were provided twice weekly for 1.5 hours each to prepare patients for reintegration into the workforce. Nurses led the behavioral and skill rehabilitation sessions, tailoring interventions to meet the individual needs of patients. They monitored patients’ engagement with these activities, assessed progress, and adjusted the program as needed to maximize rehabilitation outcomes, ensuring optimal skill development and readiness for social reintegration.
3. Social Skills Training: For patients with limited social abilities, individualized and group sessions were offered to enhance interpersonal skills. Activities included role-playing, peer discussions, and social adaptation exercises to promote social functionality. Nurses took a leading role in conducting social skills training, organizing both individual and group sessions to improve patients’ interpersonal interactions.
4. Family-Based Support: Monthly family education sessions covered patient care, medication management, and maintenance treatment. Family members were encouraged to provide understanding and avoid stigmatizing behaviors, thus fostering a supportive home environment. Nurses facilitated the family education sessions, empowering family members with the necessary knowledge and resources to support patients at home. They provided guidance on how to manage medication, reduce stigma, and maintain a supportive environment, which was crucial for improving patients’ adherence and quality of life.
5. Medication Supervision: Monthly sessions for patients and families included medication management, identification of side effects, and the importance of adherence. For those experiencing significant side effects, dosage adjustments were discussed, aiming to improve compliance and minimize patient concerns. Nurses independently monitored medication adherence, identified potential side effects, and provided individualized interventions. They facilitated discussions with patients and families regarding any adverse effects, provided reassurance, and worked closely with the prescribing physician to adjust dosages when necessary to optimize treatment outcomes.

### 2.4 Data collection and outcome measures

Relapse Rate and Medication Adherence: The relapse rate and medication adherence were assessed one year after implementing different nursing interventions for both groups. Medication adherence was classified into three levels: complete adherence (patients consistently follow prescribed medication schedules), partial adherence (patients occasionally forget doses or initially refuse but correct behavior after education), and non-adherence (patients refuse medication despite education, sometimes requiring enforced compliance). Overall adherence was defined as the sum of complete and partial adherence rates.

Social Functioning Recovery: Social functioning was evaluated before and six months after the interventions using the Social Disability Screening Schedule (SDSS). This tool assesses ten domains, including social skills, daily living abilities, and vocational capacity, with scores from 0 to 2. A total score above 2 indicates functional impairment, with higher scores denoting more severe social dysfunction.

Psychiatric Symptom Severity: The Brief Psychiatric Rating Scale (BPRS) was used to measure the severity of psychiatric symptoms across 28 items. Patients were rated on a scale of 1 to 7, with higher scores reflecting greater severity of symptoms and poorer psychiatric health.

Rehabilitation Status: The Modified Rehabilitation Status Scale (MRSS) was used to evaluate dependence, social engagement, activity levels, current symptoms, and abnormal behavior across 28 items in four domains. Each item was scored on an 8-point scale (0–7), with higher scores indicating poorer rehabilitation status.

### 2.5 Statistical analysis

Statistical analyses were conducted rigorously using SPSS software (Version 27.0). Initially, data were categorized as either quantitative or categorical, followed by normality testing to determine their distribution characteristics. For quantitative data that followed a normal distribution, inter-group comparisons were performed using independent samples t-tests, with results expressed as mean ± standard deviation. Categorical variables were represented by frequencies and percentages, and their associations or independence were evaluated using Chi-square (χ²) tests. If Chi-square test assumptions were not met, Fisher’s exact test was applied. All statistical tests were two-tailed, and significance was defined as a p-value of less than 0.05.

## 3 Results

### 3.1 Patient demographics and baseline characteristics

The study enrolled 156 patients with schizophrenia, of whom 76 comprised the control group (41 males, 35 females; age 21–43 years, mean 26.58 ± 9.18 years; illness duration 1–5 years, mean 3.56 ± 1.58 years) and 80 formed the observation group (43 males, 37 females; age 22–45 years, mean 27.66 ± 9.29 years; illness duration 1–5 years, mean 3.28 ± 1.36 years). Diagnostic subtypes (DSM-5) in the control group were paranoid (n = 34, 44.7%), disorganized (n = 24, 31.6%), and residual (n = 18, 23.7%), whereas in the observation group they were paranoid (n = 36, 45.0%), disorganized (n = 25, 31.3%), and residual (n = 19, 23.7%). Baseline overall psychopathology, measured by BPRS, was comparable between groups (control: 36.90 ± 5.31; observation: 37.27 ± 5.16; t = 0.44, p = 0.660), as was social dysfunction assessed by SDSS (control: 10.47 ± 2.37; observation: 10.71 ± 2.39; t = 0.63, p = 0.530), confirming demographic, diagnostic, and symptom‐severity equivalence at baseline (**Table 1**).

**Table 1:** Baseline characteristics of control and observation groups.

Characteristic	Control group (n = 76)	Observation group (n = 80)	t/χ²-value	p-value
Sex (male/female)	41/35	43/37	0.001	0.980
Age (years), mean ± SD (range)	26.58 ± 9.18 (21–43)	27.66 ± 9.29 (22–45)	0.730	0.467
Illness duration (years), mean ± SD (range)	3.56 ± 1.58 (1–5)	3.28 ± 1.36 (1–5)	1.188	0.237
DSM-5 diagnostic subtype, n (%)			0.006	0.958
Paranoid	34 (44.7%)	36 (45.0%)	–	–
Disorganized	24 (31.6%)	25 (31.3%)	–	–
Residual	18 (23.7%)	19 (23.7%)	–	–
SDSS total score, mean ± SD	10.47 ± 2.37	10.71 ± 2.39	0.63	0.530
BPRS total score, mean ± SD	36.90 ± 5.31	37.27 ± 5.16	0.44	0.660

SDSS, Social Disability Screening Schedule; BPRS, Brief Psychiatric Rating Scale.

### 3.2 Comparison of SDSS and BPRS scores before and after intervention

**Figure 1:**
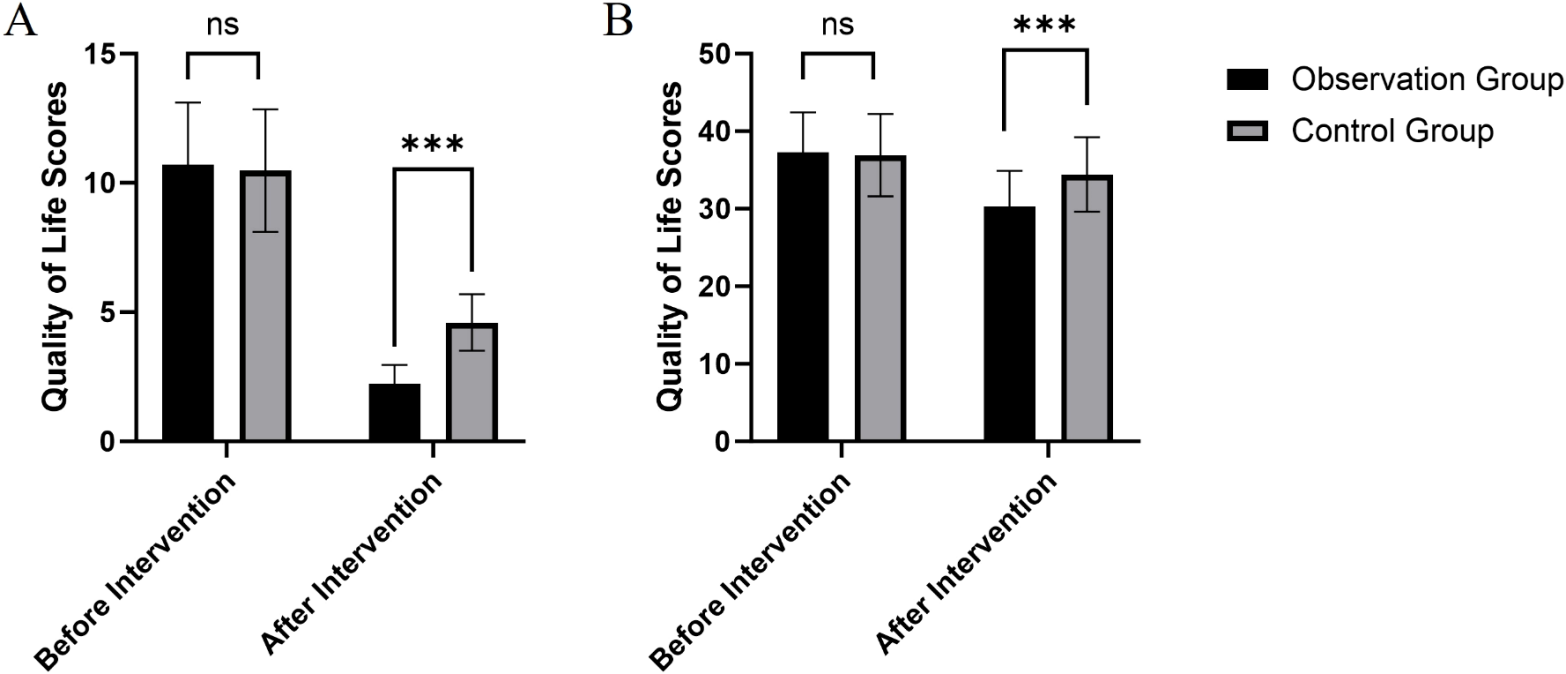
Comparison of SDSS **(A)** and BPRS scores **(B)** before and after interventions in observation and control groups. Data are presented as mean ± standard deviation. *** indicates a statistically significant difference between groups at p < 0.001. ns indicates no significant difference (p > 0.05).

Baseline SDSS and BPRS scores did not differ significantly between the observation and control groups (SDSS: t = 0.63, p = 0.530; BPRS: t = 0.44, p = 0.660), confirming comparability prior to intervention. After the intervention, the observation group demonstrated a significantly greater reduction in social disability (SDSS) than the control group (post–pre mean difference: 8.49 ± 2.50 vs. 5.87 ± 2.32; t = 16.03, p < 0.001; Cohen’s d = 2.57), reflecting a very large effect on social functionality. Similarly, psychiatric symptom severity (BPRS) decreased more in the observation group than in controls (mean change: –7.0 ± 5.23 vs. –2.49 ± 5.01; t = 5.476, p < 0.001; Cohen’s d = 0.88), indicating a large intervention effect on symptom management (**Table 2**, **Figure 1**).

**Table 2:** Comparison of pre- and post-intervention SDSS and BPRS scores between observation and control groups (Mean ± SD).

Indicator	Observation group (n = 80)	Control group (n = 76)	t-value	p-value
SDSS Score (points)				
Before Intervention	10.71 ± 2.39	10.47 ± 2.37	0.630	0.530
After Intervention	2.22 ± 0.74*	4.60 ± 1.09*	16.03	<0.001
BPRS Score (points)				
Before Intervention	37.27 ± 5.16	36.90 ± 5.31	0.441	0.660
After Intervention	30.28 ± 4.60*	34.41 ± 4.82*	5.476	<0.001

SDSS, Social Disability Screening Schedule; BPRS, Brief Psychiatric Rating Scale. * indicates significant improvement post-intervention (p < 0.05).

### 3.3 Comparison of MRSS recovery state scores between observation and control groups

Baseline comparisons indicated no significant differences between groups in dependency (t = 0.759, p = 0.449), social function (t = 0.161, p = 0.872), activity ability (t = 0.176, p = 0.861), or symptom behavior (t = 1.761, p = 0.080), confirming baseline equivalence. After the intervention, the observation group demonstrated significantly greater improvements across all MRSS dimensions compared with the control group: dependency scores decreased from 24.25 ± 3.18 to 16.30 ± 2.62 versus 23.86 ± 3.24 to 20.49 ± 2.91 (t = 9.460, p < 0.001, Cohen’s d = 1.52), social function improved from 21.05 ± 2.74 to 13.60 ± 3.15 versus 20.98 ± 2.68 to 16.30 ± 2.53 (t = 5.884, p < 0.001, d = 0.94), activity ability increased from 16.22 ± 3.15 to 11.57 ± 2.21 versus 16.13 ± 3.25 to 13.48 ± 2.87 (t = 4.671, p < 0.001, d = 0.75), and symptom behavior declined from 14.29 ± 3.10 to 8.93 ± 2.03 versus 15.17 ± 3.14 to 11.20 ± 2.11 (t = 6.848, p < 0.001, d = 1.10), indicating that evidence-based nursing interventions produced statistically significant, large to very large effects on recovery and functional status (**Table 3**).

**Table 3:** Comparison of MRSS recovery state scores between observation and control groups (Mean ± SD).

Indicator	Observation group (n = 80)	Control group (n = 76)	t-value	p-value
Dependency				
Before Intervention	24.25 ± 3.18	23.86 ± 3.24	0.759	0.449
After Intervention	16.30 ± 2.62*	20.49 ± 2.91*	9.460	<0.001
Social Function				
Before Intervention	21.05 ± 2.74	20.98 ± 2.68	0.161	0.872
After Intervention	13.60 ± 3.15*	16.30 ± 2.53*	5.884	<0.001
Activity Ability				
Before Intervention	16.22 ± 3.15	16.13 ± 3.25	0.176	0.861
After Intervention	11.57 ± 2.21*	13.48 ± 2.87*	4.671	<0.001
Symptom Behavior				
Before Intervention	14.29 ± 3.10	15.17 ± 3.14	1.761	0.080
After Intervention	8.93 ± 2.03*	11.20 ± 2.11*	6.848	<0.001

* indicates significant improvement post-intervention (p < 0.05).

### 3.4 Comparison of medication adherence between observation and control groups

The analysis of medication adherence revealed significant differences between the observation and control groups. Total adherence, which includes both complete and partial adherence, was higher in the observation group compared to the control group (62.5% vs. 65.8%, respectively). Although the proportion of complete adherence was notably higher in the observation group (32 patients) than in the control group (15 patients), partial adherence was more evenly distributed across both groups. Conversely, non-adherence was more frequent in the control group (36 patients) compared to the observation group (20 patients). Statistical analysis showed a significant association between evidence-based nursing interventions and improved medication adherence, as indicated by a χ² value of 8.474 and a p-value of 0.003. These findings suggest that structured and targeted nursing interventions positively influence patients’ compliance with prescribed medication regimens, reducing the prevalence of non-adherence and promoting better overall adherence outcomes (**Table 4**).

**Table 4:** Comparison of medication adherence between observation and control groups.

Group	Complete adherence (n)	Partial adherence (n)	Non-adherence (n)	Total adherence (n, %)
Control Group (n = 76)	15	25	36	40 (52.6%)
Observation Group (n = 80)	32	28	20	60 (75.0%)
χ² value	–	–	–	8.474
p value	**-**	**-**	**-**	0.003

## 4 Discussion

Schizophrenia is a chronic psychiatric disorder with persistent symptoms and significant functional impairment, presenting challenges in clinical management despite pharmacological advances (18, 19). EBN interventions, which integrate clinical expertise and research evidence, are increasingly recognized for their role in improving outcomes in this population (20, 21). The results of this study highlight the effectiveness of EBN interventions in managing schizophrenia, particularly in enhancing social functionality, psychiatric symptom management, patient recovery states, and medication adherence. The novelty of this study lies in its comprehensive, multidisciplinary approach, which addresses not only the psychiatric symptoms but also the social and functional impairments that are central to the lives of patients with schizophrenia. Clinically, the findings underscore the substantial improvements in both social functioning and medication adherence, suggesting that EBN interventions are not only effective but also necessary for improving long-term treatment outcomes. The clinical application of these findings is significant, as they provide a structured framework for enhancing patient engagement, reducing relapse rates, and improving overall quality of life in schizophrenia care. By incorporating EBN principles into routine clinical practice, healthcare providers can foster a more holistic and patient-centered treatment model that improves both psychological and functional outcomes for patients with schizophrenia.

The significant reduction in SDSS scores in the observation group (from 10.71 to 2.22, p < 0.001) indicates substantial improvement in social functionality, suggesting that EBN interventions effectively address social deficits in patients with schizophrenia. This improvement is likely due to the integration of tailored psychosocial support and skill-building activities, such as social skills training and cognitive-behavioral techniques. These strategies may have helped patients regain confidence in social interactions, improve interpersonal skills, and adapt to societal norms, thereby facilitating their reintegration into the community. Similarly, the greater reduction in BPRS scores in the observation group (from 37.27 to 30.28, p < 0.001) highlights better psychiatric symptom control. EBN interventions, emphasizing consistent psychoeducation and emotional support, may have enhanced patients’ understanding of their condition, reduced stigma, and improved treatment adherence. Moreover, the structured and systematic approach of EBN likely contributed to the timely identification and management of symptom exacerbations, preventing symptom escalation and improving overall clinical outcomes (22, 23).

The observation group demonstrated superior outcomes across all MRSS indicators, including dependency (from 24.25 to 16.30, p < 0.001), social function (from 21.05 to 13.60, p < 0.001), activity ability (from 16.22 to 11.57, p < 0.001), and symptom behavior (from 14.29 to 8.93, p < 0.001). The significant reduction in dependency scores highlights the role of EBN in promoting patient autonomy, likely due to individualized care plans and goal-oriented rehabilitation exercises that empower patients to manage daily activities independently. The marked improvement in social function and activity ability underscores the value of interventions aimed at enhancing interpersonal skills and encouraging participation in meaningful activities. Vocational counseling, role-playing, and structured tasks provided patients with opportunities to practice and develop these skills in a supportive environment (24, 25). Furthermore, reductions in symptom behavior suggest that EBN interventions effectively addressed underlying psychological and behavioral challenges, leading to better emotional regulation and fewer disruptive symptoms.

The analysis of medication adherence revealed significant benefits of EBN interventions. The observation group demonstrated a higher rate of complete adherence (40%) compared to the control group (19.7%), which can be attributed to comprehensive medication education, routine monitoring, and individualized support. These interventions likely addressed common adherence barriers, such as misunderstanding the importance of consistent medication use and concerns about side effects. By providing clear instructions and ongoing encouragement to patients and their families, EBN interventions helped mitigate these challenges and fostered trust in the treatment process (26, 27). The lower rates of non-adherence in the observation group (25.0% vs. 47.4% in the control group) highlight the role of structured nursing practices in improving compliance. Techniques such as motivational interviewing and problem-solving likely contributed to this improvement by helping patients overcome treatment resistance and reinforcing positive behaviors. This result is particularly significant given the strong association between medication adherence and long-term treatment outcomes in schizophrenia.

Several mechanisms may underlie the observed benefits of EBN interventions in schizophrenia care. First, the focus on individualized care allows for the tailoring of interventions to each patient’s specific needs, thereby enhancing clinical relevance and therapeutic efficacy. Second, the systematic application of evidence-based protocols ensures consistency and standardization in nursing practices, minimizing care variability and promoting more reliable outcomes. Third, the incorporation of multidisciplinary collaboration within the EBN framework strengthens the comprehensiveness of care by simultaneously addressing pharmacological, psychosocial, and behavioral dimensions of the disorder. Psychoeducation plays a particularly critical role by improving patients’ insight into their condition and treatment, thereby reducing internalized stigma, increasing motivation, and fostering active engagement in care (28, 29). This is further supported by structured behavioral interventions, which equip patients with practical skills for managing symptoms and improving social functioning. Several limitations must be acknowledged. First, the retrospective design introduces potential selection bias, which may limit the generalizability of the findings. Additionally, reliance on self-reported adherence data could introduce bias in assessing medication compliance. Second, intervention fidelity was not formally monitored. Although nurses followed standardized protocols and received training, the absence of fidelity tracking tools (e.g., session checklists, supervision logs, or inter-rater reliability measures) may have led to inconsistencies in intervention delivery. Third, outcome assessments were not blinded, as the same clinical team that delivered the interventions also collected post-intervention data, introducing a risk of observer bias. To address these methodological limitations, future research should adopt prospective or randomized controlled designs with concealed allocation, blinded assessment, and structured fidelity monitoring to ensure consistent and unbiased delivery of intervention components. In addition, incorporating objective measures of adherence—such as pill counts, electronic monitoring, or pharmacy refill data—would improve the accuracy of outcome evaluation. Furthermore, we acknowledge the advantages of quasi-experimental or cluster-randomized designs in clinical environments, which can better account for organizational and contextual confounders. Finally, although cost-effectiveness is a critical consideration for clinical implementation, our study was unable to conduct a formal economic evaluation due to limitations in retrospective data and billing records, which did not capture nursing-specific costs. Future research should explore the economic impact of EBN to support informed policy and resource allocation decisions. Overall, while this study provides robust evidence for the effectiveness of EBN interventions, future studies with enhanced methodological rigor will be instrumental in validating and scaling EBN strategies in schizophrenia care.

## 5 Conclusions

This study demonstrates that evidence-based nursing interventions significantly enhance social functionality, psychiatric symptom management, recovery states, and medication adherence in patients with schizophrenia. The results highlight the value of structured, evidence-informed nursing practices in addressing the multifaceted challenges of schizophrenia, emphasizing their role in optimizing clinical outcomes and promoting holistic patient recovery.

## Data Availability

The raw data supporting the conclusions of this article will be made available by the authors, without undue reservation.
